# Seed predation and potential seed dispersers of the narrow endemic *Ceratozamianorstogii* (Zamiaceae)

**DOI:** 10.3897/BDJ.10.e86007

**Published:** 2022-08-24

**Authors:** Héctor Gómez-Domínguez, Jessica E Hernández-Tapia, Andrés E. Ortiz-Rodriguez

**Affiliations:** 1 Senda sustentable, AC, Berriozábal, Chiapas, Mexico Senda sustentable, AC Berriozábal, Chiapas Mexico; 2 Departamento de Botánica, Instituto de Biología, Universidad Nacional Autónoma de México (UNAM), Ciudad de México, Mexico Departamento de Botánica, Instituto de Biología, Universidad Nacional Autónoma de México (UNAM) Ciudad de México Mexico

**Keywords:** Cycads, kinkajou, Mexico, reproductive ecology, rodent dispersal, skunk

## Abstract

In this study, we report the observation of potential seed dispersers of the endemic to Mexico and narrowly distributed *Ceratozamianorstogii* (Zamiaceae). Camera traps were installed in front of two plants of *Ceratozamianorstogii* and cone phenology until their maturity and disintegration was determined. The female cone of *Ceratozamianorstogii* has a development of ten months, from the time it emerges until it disintegrates. We were able to identify three stages of cone development: 1) Pre-pollination phase, 2) Pollination phase and 3) Seed maturation phase. Our results support an animal-dispersal hypothesis in *Ceratozamia*. Three mammals [a mouse (*Pteromiscus* sp.), a southern spotted skunk (*Spilogaleangustifrons*) and a kinkajou (*Potusflavus*)] were recorded biting, carrying or removing seeds of *Ceratozamianorstogii*. The camera traps recorded no evidence of birds or other mammals coming to the cones to feed. Thus, interaction of frugivores with seeds occurs at night. The most frequent visitor was the mouse, followed by the southern spotted skunk and the kinkajou. Significant differences (GLM, p< 0.05) in visitor frequency and time for interaction were found between species. We believe that the mouse is probably the most effective seed disperser for *Ceratozamianorstogii*. The results presented here have evolutionary implications that can be scaled to the entire genus *Ceratozamia*. Specifically, short-distance dispersal promotes allopatric speciation in this group of plants.

## Introduction

*Ceratozamia* (Zamiaceae), with ~ 35 species is one of the most diverse gymnosperms in Mexico (Martínez-Domínguez et al. 2021). All species of *Ceratozamia* (except *Ceratozamiarobusta*) are endemic to Mexico, so the genus diversification is linked to the evolutionary history of this region, specifically with the complex climatic history of northern Mesoamerica (from Mexico to Nicaragua) (Medina-Villarreal et al. 2019, Gutiérrez‐Ortega et al. 2020). In recent years, the taxonomy and distribution of *Ceratozamia* species have been widely studied and many aspects of their evolutionary history are now understood (Vovides 2000, Nicolalde-Morejón et al. 2014). However, little is known about its reproductive ecology (Martínez-Domínguez et al. 2018a, Martínez-Domínguez et al. 2021 and references therein).

Some authors suggest that cycad species, in general, can be dispersed by rodents (González-Christen 1990,Yáñez-Espinosa et al. 2021), birds (Eckenwalder 1980, Velazco-García et al. 2016) and small and medium-sized mammals (Pérez-Farrera and Vovides 2004,Velazco-García et al. 2016 ,Monteza‐Moreno et al. 2022). This seed-animal interaction could be unexpected in cycads because their seeds produce toxic substances (mainly methylazoxymethanol glycosides) that, when ingested, cause symptoms of poisoning in vertebrates (Moretti et al. 1983, Hall and Walter 2014a). In the particular case of the genus *Ceratozamia*, it is believed that gravity plays an important role in seed dispersal (Martínez-Domínguez et al. 2021), but some observations of peccaries consuming seeds of *Ceratozamiamatudae* Lundell and *C.mirandae* Vovides, Pérez-Farr. & Iglesias (Pérez-Farrera et al. 2000,Pérez-Farrera and Vovides 2004, Pérez-Farrera et al. 2006), and seeds inside burrows (Martínez-Domínguez et al. 2018a,Martínez-Domínguez et al. 2018b,Monteza‐Moreno et al. 2022, Martínez-Domínguez et al. 2018a) suggests that this generalization for the genus is wrong. In addition, the large seeds of *Ceratozamia*, visually conspicuous and covered by a fleshy and brightly coloured sarcotesta, which also release a sweet, pungent odour, supports an animal-dispersal hypothesis (Yang et al. 2012,Hall and Walter 2013 ,Hall and Walter 2014b ,Martínez-Domínguez et al. 2018a)

Here we report the observation of seed predators and potential seed dispersers of the endemic to Mexico and narrowly distributed *Ceratozamianorstogii* D.W. Stev. (Zamiaceae). Specifically, we show the results of ten months of observation on strobili of this species. Our results aim to contribute to the knowledge about the reproductive ecology of this important group of plants, where most of its species are endangered (https://www.iucnredlist.org/search?query=Ceratozamia&searchType=species).

## Methods

### Study system

*Ceratozamianorstogii* is a species endemic to Mexico restricted to the Pine-oak forests and cloud forests in southern Mexico, in the states of Chiapas and Oaxaca. Individuals of this species have underground trunks 12 to 130 cm long, with a crown of 15 or more long-pinnate fronds at the tip, the fronds between 60 and 140 cm in length (Pérez-Farrera et al. 2001, Fig. 1). In this species of *Ceratozamia*, the very narrow leaflets are spirally distributed along the rachis and have small spines on the margin that decrease in frequency towards their base, these being a distinct characteristic of the species (Stevenson 1982). *Ceratozamianorstogii* is dioecious, and produces either a single pale-yellow or cream polleniferous strobilus (male cone) or a single grayish-green to dark-brown ovulated strobilus (female cone). Male cones are conical, thinner towards the tip and can measure from 25 to 36 cm in length and from 3.8 to 5 cm in diameter (Pérez-Farrera et al. 2001), whilst the female cones are cylindrical, as long as wide, 21-37 cm long and 9-13 cm in diameter and with more than 100 angulars to ovoid, arylated seeds, 2.4-3 cm long and 1.5-2 cm in diameter. When mature, the aril on the seeds (sarcotesta) changes colour from yellow to brown and releases a sweet, pungent odour (Pérez-Farrera et al. 2001). A hard seed coat, odour release, and striking colour are characteristics that suggest animal seed dispersal, but so far, no animal dispersers have been observed in *Ceratozamianorstogii*. Overexploitation and land-use change threaten the populations of *Ceratozamianorstogii* [Endangered under criteria A2abd; B1ab (iii, iv, v)].

### Data collecting

For ten months (October 2020 to July 2021), two camera traps (Bushnell prime 24 megapixelles low glow) were installed in front of two female plants of *Ceratozamianorstogii* with the objective of determining their cone phenology until maturity and disintegration and identifying predators and potential seed dispersers. The two plants were located within a mature forest far from the edge, roads or crops. At the site, the density of individuals was high, but most were sterile, so each plant was separated from the other by at least 50 meters. We measured and recorded the changes in size and colouration of the cones at each stage of their development. Once the seeds mature, diurnal and nocturnal foraging activity, including frequency of visits and time for interaction were recorded (supplementary materials). The observations were carried out within the "La Sepultura" Biosphere Reserve in Chiapas, Mexico (the exact location of the population is not declared considering the vulnerability of this species).

### Data analyses

We used a poisson regression approach for handling the count data. We performed two generalized linear models (glm) using the frequency of visits per night and the time of interaction per night as response variables and each frugivore as a factor [*glm (variable ~ Species, family = "poisson")*]. For each analysis, we performed an Analysis of Deviance to determine differences within species (chi-square test, Fox and Weisberg 2019). Then, we carry out a Least-squares means test to assess differences between pairs of species (tukey *p*-adjust), using the *emmeans* function (Lenth 2022). A higher frequency of visits with prolonged times of activity on the seeds (e.g. biting, carrying or removing the seed coat) was considered as evidence of potential seed dispersed. All analyses were done with R software (R Core Team, 2020).

## Results

### Phenology

**Figure 2.**
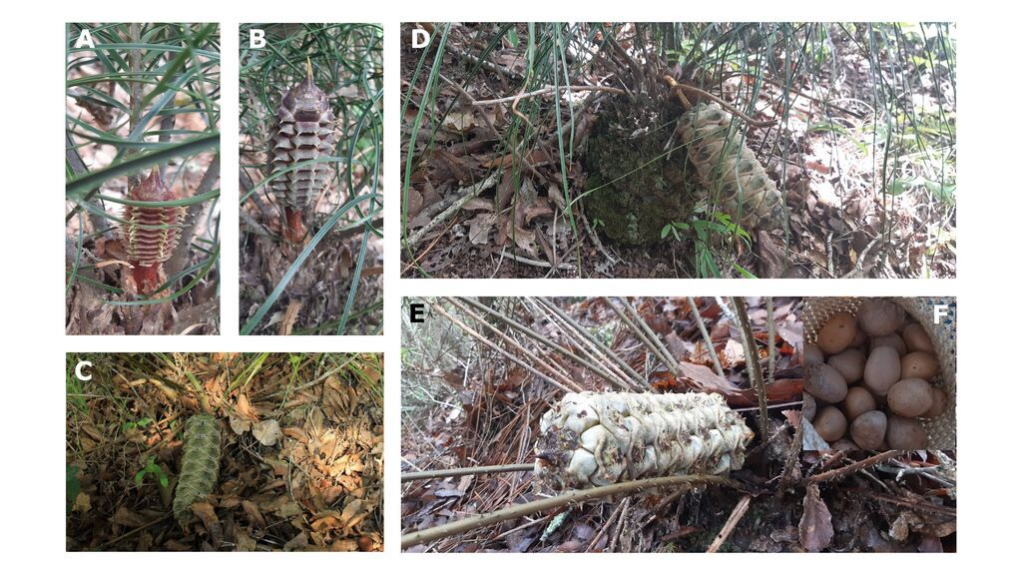
Female cone maturation. **Pre-pollination phase**. **A** Emergent cone, with a short, straight peduncle and a general reddish-brown colouration **B** Young cone, a larger brown cone with a straight peduncle **C** a large, fully developed cone with a greenish colouration, peduncle much longer and bent towards the ground. **Pollination phase**. **D** A large, pendant cone, with a light brown colouration, and barely separation amongst megasporophylls. **Seed maturation phase**. **E** A large, pendant cone, with a light brown colouration and with an evident separation amongst megasporophylls. **F** Mature seeds. Photos by Ana G. Rocha.

The female cone of *Ceratozamianorstogii* has a development of ten months, from the time it emerges until it disintegrates. In the population studied here, this period includes the months between October 2020 and July 2021. We were able to identify three stages of cone development (Fig. 2): **A** Pre-pollination phase [seven months (Oct.-April.)]: this phase includes the first stages of development of the cone until it reaches its maximum size. Emerging and young cones (50─110 by 30─40 mm) have a reddish-brown surface with greenish or yellow-coloured spine tips, and erect, short peduncles (50 to 100 mm long). As they grow the cones turn grayish-green in colour, increase in length and width (110─240 by 40─80 mm), their peduncles are longer (up to 120 mm) and bend progressively towards the ground. The phase concludes with a change in the surface color of the cones, which appear completely decumbent or pendent **B** Pollination phase [around two months (May.-Jun)]: this phase is characterized by the receptivity of the cone (a barely separation amongst megasporophylls, a yellow-amber liquid exuding and a sweet, pungent odour) accompanied by the recurrent presence of medium-size beetles (Coleoptera: Erotylidae: Pharaxonothinae) as specific pollinators. Also in this phase, the general colour of the cone surface may change from a grayish-green colour to a light brown or dark brown colour. **C** Seed maturation phase [around three months (Jun-Aug.)]: in this phase, the separation amongst megasporophylls is more evident and seeds change colour progressively from yellow to brown. The cones continue to produce a liquid exudate and odour release is also maintained. In this phase, where the visit of frugivores, takes place, which bite and carry seeds until the total disintegration of the cone.

### Diversity of visitors

**Figure 3.**
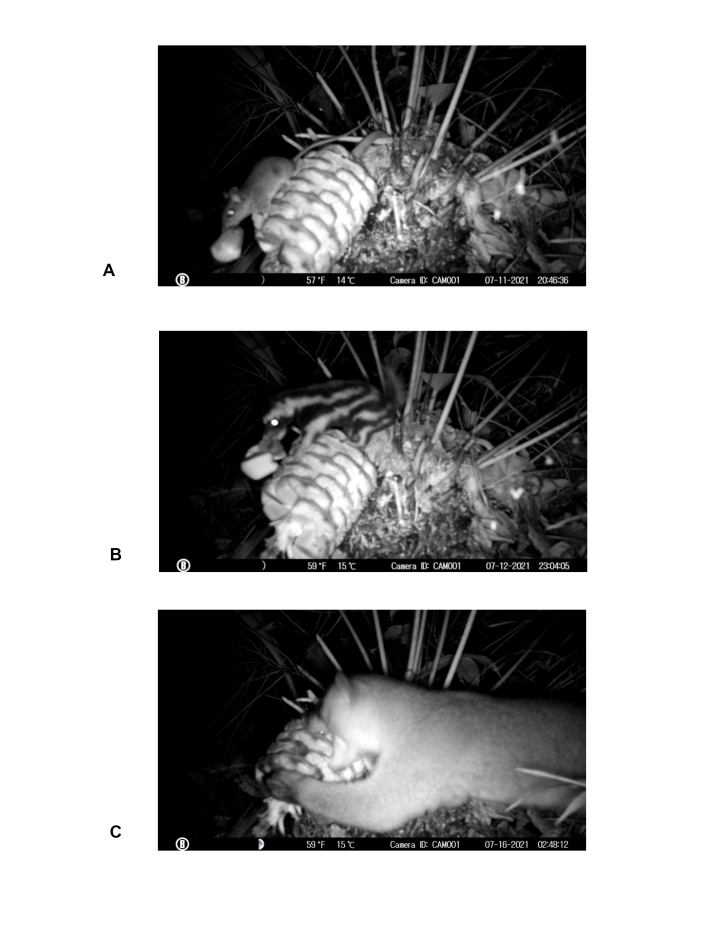
Female cone visitors in *Ceratozamianorstogii.* **A** *Pteromiscus* sp. (Mouse) collecting seeds; **B** *Spilogaleangustifrons* (a southern spotted skunk) biting the cone and collecting seeds; **C** *Potusflavus* (Kinkajou) removing seeds.

During the 10 months, camera traps captured seven visitors to the female cone of *Ceratozamianorstogii* (Table 1). The highest number of observations was recorded during the night hours (nocturnal visitors) and less frequently during sunny hours (diurnal visitors). Observations were recorded both at young stages of the cone and at mature stages of its development (seed maturation phase). During sunny hours, three bird species were recorded using the cone as a perch, two on an immature cone and the other on the remains of a disintegrated cone. Also in daylight, a badger stopped for a moment to smell the cone and then continued on its way. During the night, the visitors were small and medium-sized mammals (Fig. 3), which interacted directly with the cone. A mouse (*Pteromiscus* sp.) was frequently observed feeding on the cone exudat and carrying seeds beyond the visible range of the camera (~ 2 m). A kinkajou (*Potusflavus* was observed visiting the cone and removing the seeds and biting the central axis of the cone. A southern spotted skunk (*Spilogaleangustifrons)* was very active and was observed biting and carrying seeds beyond the visible range of the camera on several occasions (Table 1, Fig. 3).

### Frugivores foraging activity and potential seed dispersers

**Figure 4.**
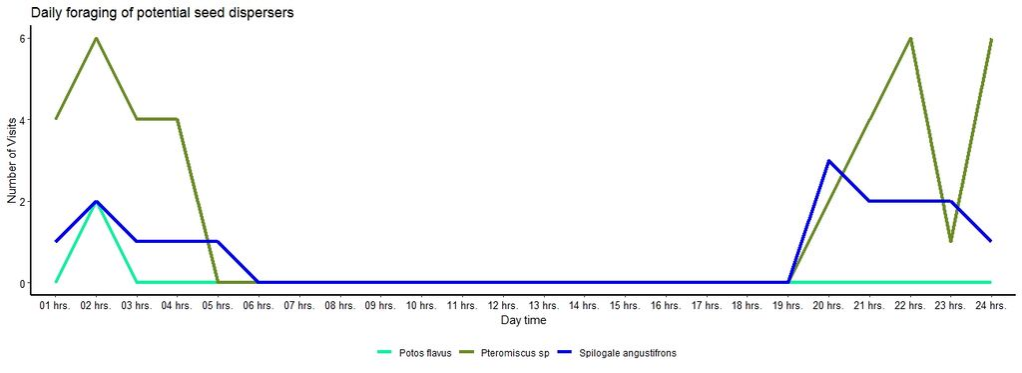
Nocturnal foraging activity of potential seed dispersers.

**Figure 5.**
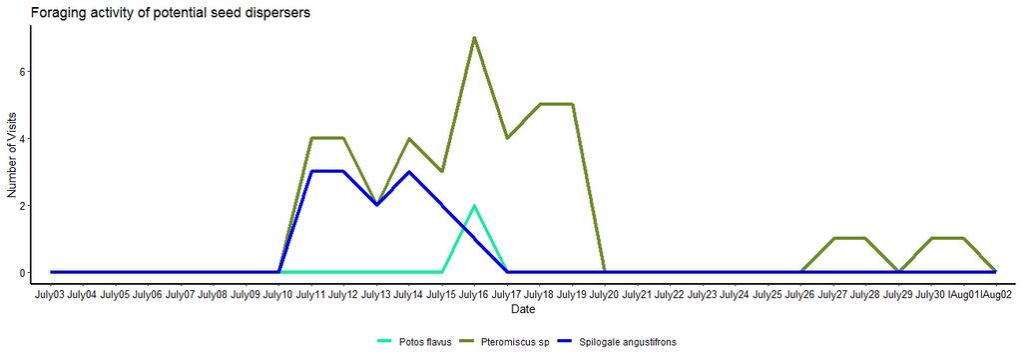
Foraging activity and potential seed dispersers.

**Figure 6.**
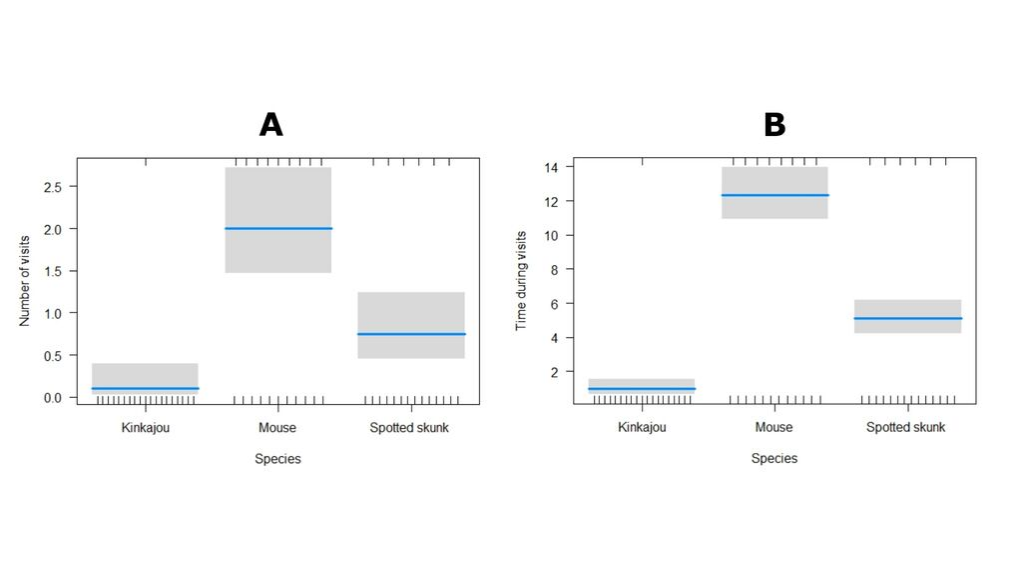
Total number of visits and visits duration per night. **A** ;The fitted number of visits (in original scale) according to the results of the GLM analysis. **B** The fitted values of visit duration (in original scale = seconds) according to the results of the GLM analysis.

Three mammals (a mouse, a southern spotted skunk and a kinkajou) were observed biting, carrying or removing seeds of *Ceratozamianorstogii* (Table 1, Fig. 3). Interaction of frugivores with seeds occurs at night (after eight at night). Frugivore activity increases between midnight and three in the morning (Fig. 4) and it is during the second week of July that frugivore activity was most frequent (Fig. 5). Activity was observed for 20 nights, with visitors observed on 65% of them, with an overall estimate of three visits per night (57 visits in total). The mouse of the genus *Pteromiscus* was observed frequently during a large part of the seed maturation phase [13 nights (65% of total observation nights), 40 visits in total, mean time per interaction = 6.1 ± 3.1 seconds, accumulated time = 427 seconds], followed by the southern spotted skunk [6 nights (30% of total observation nights), 15 visits, mean time per interaction = 6.8 ± 3.4 seconds, accumulated time =102 seconds] and the kinkajou (one night, two visits, mean time per interaction =10 seconds, accumulated time = 20 seconds) (Fig. 4, Fig. 5). Significant differences (*tukey* pairwise comparisons, P< 0.05, Tables 2, 3) in number of visits and time for interaction were found between the three species (Fig. 6). Accordingly, the mouse can be considered here as the most effective potential seed disperser of *Ceratozamianorstogii*

## Discussion

Our results support the animal-dispersal hypothesis in *Ceratozamia*. Three small mammals, the mouse, southern spotted skunk and the kinkajou, are reported here as potential seed dispersers of *Ceratozamianorstogii*. Mice as dispersers have also been observed in other species of cycads (González-Christen 1990,Snow and Walter 2007,Yáñez-Espinosa et al. 2021), but the spotted skunk and the kinkajou biting and carrying cycad seeds are recorded for the first time. Our results showed that the mouse of the genus *Pteromiscus* was a frequent visitor to the female cone of *Ceratozamianorstogii* and was observed biting and carrying seeds sarcotesta for several nights and for a long time, even when the cone was disintegrated and the seeds scattered on the ground (Figs 4, 5, 6,Suppl. materials 1, 2). We believe that the mouse is probably the most effective seed disperser for this cycad species (Tables 2, 3). Unlike the mouse, the other two species were less frequent visitors and do not focus exclusively on the seeds. Specifically, the kinkajou aggressively removed many seeds to concentrate on the central axis (Suppl. material 1). It is likely that the female cone of *Ceratozamianorstogii* represents a seasonal food resource for the southern spotted skunk and the kinkajou. The idea that female cones represent an occasional food resource for the spotted skunk and the kinkajou is supported by the behaviour most commonly reported for both species. The southern spotted skunk and the kinkajou are small mammals that feed mainly on insects, but as omnivores, they can also feed on other smaller animals, carrion, fruits, and seeds (Kinlaw 1995). The kinkajou is a mammal considered arboreal, so its participation in the removal of *Ceratozamianorstogii* seeds with the cone at ground level is a novel finding. Southern spotted skunk species and the kinkajou are not recognized as important seed dispersers (Willson 1993). However, our results show that the southern spotted skunk can become an occasional, important, seed disperser of *Ceratozamianorstogii* (Fig. 6,Tables 2, 3). The southern spotted skunk has a longer home range than the mouse, thus increasing the chance that seeds will move a greater distance (Lesmeister et al. 2009). All cone visitors recorded here are nocturnal animals, and the removal and dispersal of *Ceratozamianorstogii* seeds occur at night. Nocturnal seed dispersal has been observed in other cycads (Hall and Walter 2013, [Bibr B8010028][Bibr B7837796]), so it is likely that nocturnal dispersal is common amongst *Ceratozamia* species.

The low diversity of visitors reported here is consistent with other studies focused on cycads, where seed removal is carried out by a low diversity of small mammals, almost specifically (Burbidge and Whelan 1982,Snow and Walter 2007,Hall and Walter 2013,Yáñez-Espinosa et al. 2021,Monteza‐Moreno et al. 2022). In addition, most of the species that interact with the cone represent sporadic visits (Burbidge and Whelan 1982,Snow and Walter 2007). In this study, visits were recorded on 65% of observation nights with an average of three visits per night. The mouse was present on all the nights where visits were observed, but the southern spotted skunk in half the nights and the kinkajou only in one night. Of the 57 total visits registered, the mouse visited the cone in 70% of them, the southern spotted skunk in 26% and the kinkajou in only 3% of the visits registered. The low and sporadic seed cone visitation rates in *Ceratozamianorstogii* suggest that most seeds do not disperse away from the parent plant, however, when they are dispersed, due to the size and behaviour of the dispersers, the seed moves only a few meters from the mother source (Tang 1989,Snow and Walter 2007,Hall and Walter 2013). The foregoing is supported by the high density of plants recorded in the study area (more than 1000 plants in one hectare), where they are also distributed in a gregarious manner (Martínez-Meléndez 2012). The spatial arrangement and density of cycad populations, with dense spatial aggregations of seeds and seedlings, could suggest dispersal by gravity. However, our results suggest an effect of short-distance seed dispersal mediated by small mammals (Burbidge and Whelan 1982,Snow and Walter 2007,Hall and Walter 2013,Yáñez-Espinosa et al. 2021). It is important to recognize that our study is limited to a sample size of two plants, which implies the possibility of not recording the full diversity of potential seed dispersers. However, the diversity of dispersers in *Ceratozamianorstogii*as, as in other cycads, should not be much greater.

The results presented here have evolutionary implications that can be scaled to the entire genus *Ceratozamia*. Specifically, short-distance dispersal promotes allopatric speciation in this group of plants. The above could be accentuated in periods of climatic change, such as those that occurred in Mexico during the Miocene and Holocene. According to the divergence time estimates for the genus (Condamine et al. 2015,Medina-Villarreal et al. 2019), these are the epochs with the highest pulses of speciation within *Ceratozamia*. Some authors suggest that short-distance dispersal in cycads may arise as a result of the extinction of large mammals capable of dispersing massive fruits (Hall and Walter 2013). Moreover, most of the speciation events in *Ceratozamia* occurred after the large mammals of North America became extinct or when their populations were in decline. Under this hypothesis, it is likely that ancient large mammals dispersed the *Ceratozamia* seeds from one place to another, connecting distant populations and, thus, diluting the effects of distance and isolation. These mammals were probably tolerant to the toxins present in cycad seeds (Hall and Walter 2014). In this scenario, small mammals, such as the mouse, could act as secondary seed dispersers. As large mammals became extinct, the role of small mammals gained greater relevance, affecting the connectivity between populations and promoting higher spatial density in each of them.

## Conservation implications

Most *Ceratozamia* species have small ranges with allopatrically distributed sub-populations. Based on the results obtained here, asmall mammal dispersal is occurring, implying a limited gene flow between spatially-isolated sub-populations. Thus, knowing the mechanisms and modes of dispersal of these cycads provides valuable information to better plan conservation strategies, such as germination or propagation work.

## Supplementary Material

98794683-7C28-5E67-9530-123122B92C1C 10.3897/BDJ.10.e86007.suppl1 Supplementary material 1 Table S1. Data type Species activity observations Brief description A database that includes dates, the identity of the species, visits by observation and the time that each visit lasted. File: oo_712769.csv https://binary.pensoft.net/file/712769 Gómez-Domínguez et al.

33E09CBF-0F49-540D-8A34-BD52AA82F0B6 10.3897/BDJ.10.e86007.suppl2 Supplementary material 2 Table S2. Visit per night and total interaction time per night Data type Species activity observations Brief description A condensed database that includes dates, species identities, observation visits per night and the total interaction time per night. File: oo_712878.csv https://binary.pensoft.net/file/712878 Gómez-Domínguez et al.

## Figures and Tables

**Figure 1.**
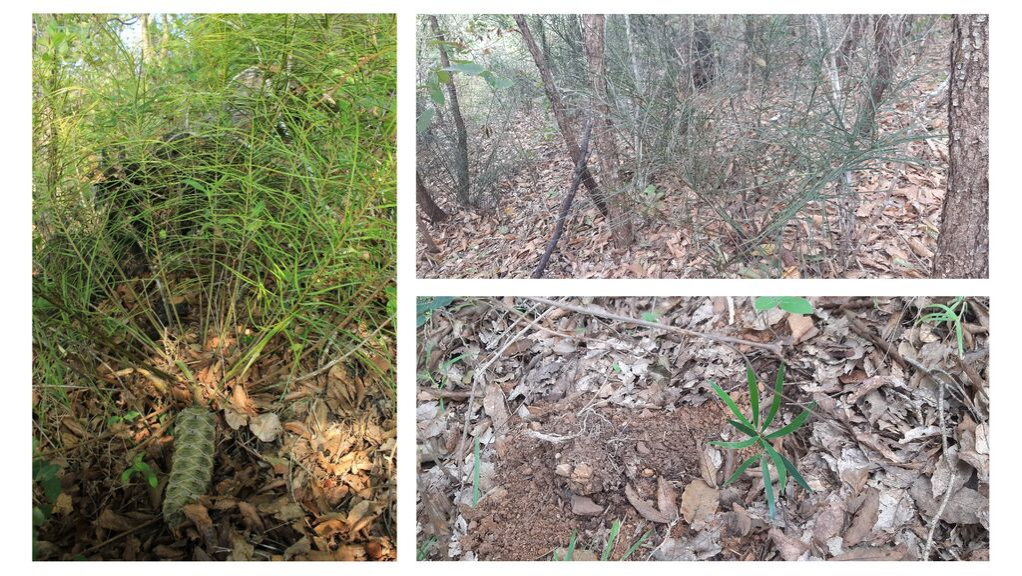
*Ceratozamianorstogii* in the study area. On the left, plant in reproductive phase (pre-pollination). Top right, habitat (pine-oak forest). On the bottom right, a seedling growing amongst the leaf litter. Photographs by Héctor Gómez Domínguez and Ana G. Rocha.

**Table 1.** Cone visitors in *Ceratozamianorstogii*. The activity period includes diurnal and nocturnal. The diurnal visit covers from five in the morning until eight at night. The nocturnal visit covers from eight at night until five in the morning. The general behaviour of each visitor is reported.

**Species**	**Period of activity**	**General behaviour**
*Momotusmexicanus* (russet-crowned motmot)	Diurnal	Perched
*Basileuteruslachymosa* (fan-tailed warbler)	Diurnal	Perched
*Spilogaleangustifrons* (Southern spotted skunk)	Nocturnal	Bites and takes some seeds, walks around
*Pteromiscus* sp. (Mouse)	Nocturnal	Bites and takes some seeds, walks around
*Nassuanarica* (white-nosed coati)	Diurnal	Sniffs the cone and walks away
*Potusflavus* (Kinkajou)	Nocturnal	Bites some seeds, walks around

**Table 2.** Summary of the GLM results using the number of visits as a response variable and the species as predictors. *p* values < 0.001 ***, p < 0.01 **, and p < 0.05 *

**Variable**	**Coefficient**	**SE**	**z value**	**p**
Number of visits				
Observations = 60				
Intercept	-2.3026	0.7071	-3.256	**
Mouse	2.9957	0.7246	4.135	***
Spotted skunk	2.0149	0.7528	2.677	**
**Species pairwise comparisons**				
Kinkajou - Mouse ***				
Kinkajou - Spotted skunk *				
Mouse - Spotted skunk ***				

**Table 3.** Summary of the GLM result using the time during the visit as a response variable and the species as predictors. *p* values < 0.001 ***, p < 0.01 ** , and p < 0.05 *

**Variable**	**Coefficient**	**SE**	**z value**	**p**
**Time during visits**				
Observations = 60				
Intercept	3.62E-08	2.24E-01	0	
Mouse	2.51E+00	2.33E-01	10.814	***
Spotted skunk	1.63E+00	2.45E-01	6.663	***
**Species pairwise comparisons**				
Kinkajou - Mouse ***				
Kinkajou - Spotted skunk ***				
Mouse - Spotted skunk ***				
